# Supplementary material for: On the Accuracy of Language Trees
Source: PLoS One. 2011 Jun 3;6(6):e20109. doi: 10.1371/journal.pone.0020109 (PMC3108590; doi:10.1371/journal.pone.0020109)
Supplement: File S1 — (PDF) [file pone.0020109.s001.pdf]

Supporting Information for

On the accuracy of language trees by Pompei et al.

1 Analysis of ASJP database and trees inference

Here we discuss in details some quantitative features of the ASJP and Ethnologue databases. Table S1 summarizes, for each language family in the ASJP database, the number N of languages, the corresponding resolution (as defined below) of the Ethnologue classification, and two properties of words lists discussed in the main text: M_{eff} and the Coverage. Histograms showing the distribution across all the language families of the same quantities are shown in figure S1. We note that, while M_{eff} and Coverage are nearly constant for all the language families in the ASJP database, the number N of languages and the Ethnologue resolution feature a great variability. We quantify the resolution of the Ethnologue classification as $\frac{I-1}{N-2}$, where I is the number of internal nodes in the classification tree and N is the number of leaves. With this definition, completely unresolved classifications, i.e., star trees, will result in a null resolution, while the resolution is equal to one for complete binary classifications. These are the values shown with a color code in the main text.

For the sake of completeness, we recall the definition of M_{eff} and Coverage. The former is defined as follows: we name $f_{meaning}$ the fraction of languages in each family which contain a word for *meaning*; M_{eff} is simply the sum of $f_{meaning}$ over all the meanings expressed by a word in at least a language in the considered family. The Coverage is a quantitative measure of the degree of overlap between the lists of words of pairs of languages, defined as:

$$\text{Coverage} = \frac{2}{N(N-1)} \sum_{i \neq j} \sum_{a} l_i^a l_j^a,$$

where we define each language i as a binary vector $\vec{l_i}$, its generic entry l_i^a being 1 if a word exists in that language for the meaning a and 0 otherwise, and the sum is over all pairs of languages in the considered family.

We are now interested in analysing how these quantities affect the phylogeny reconstruction accuracy. To this end, we need to consider how the different measures of misclassification are in turn affected by the characteristics of the databases.

We then consider the accuracy of the inferred trees as measured respectively by the Robinson-Foulds distance, the Quartet Distance, the generalized Robinson-Foulds score and the generalized Quartet Distance. In particular, we investigate how the accuracy of the inferred trees is affected by the quantities considered above, namely the number N of languages in the considered family, the resolution of the corresponding Ethnologue classification, the M_{eff} and the Coverage. In tables S2 and S3 we show the Pearson correlation coefficient (also known as Pearson's r) between the distance of the inferred trees from the Ethnologue classification, as computed with the different criteria we proposed, and the quantities discussed above. In particular, table S2 shows results obtained considering the whole database while in table S3 we report results obtained by removing from the database those families with null Ethnologue resolution, i.e., for which the Ethnologue tree is a star.

In both cases, we observe a substantial difference between the standard RF and QD measures and their generalizations GRF and GQD. The standard Robinson-Foulds distance features a positive Pearson correlation with the number N of languages in a family, and both the standard Robinson-Foulds distance and Quartet Distance feature a strong negative Pearson correlation with the Ethnologue resolution. Both the GRF and GQD feature a Pearson coefficient with the Ethnologue resolution well below the

significance threshold, correcting the biases in the misclassification measure due to lack of information in the Ethnologue database (see main text).

The reconstruction accuracy does not present correlations with M_{eff} and the Coverage, the Pearson coefficient being below the significance threshold for the whole set of measures considered. However, it is important to note that this lack of correlations is actually due to the homogeneity of the ASJP data set with respect to M_{eff} and Coverage. The histograms shown in Fig. S1 for both M_{eff} and Coverage are very peaked, with small variance: this absence of variability does not allow for the detection of correlations between such parameters and the accuracy of the reconstruction. In order to overcome this limitation we performed a comparative analysis of the ASJP and the ABVD databases for the Austronesian family (presented in the main text). The usage of a new database (the ABVD database) allows for the examination of words lists with very different values for both M_{eff} and Coverage, revealing a strong dependence of the accuracy of the phylogenetic inference on such parameters.

In tables S4, S5, S6 and S7 extensive results for the accuracy of the inferred trees, as measured respectively by the Robinson-Foulds distance, the Generalized Robinson-Foulds score, the Quartet Distance and the Generalized Quartet Distance are reported. The Robinson-Foulds distance, as already stressed, is sensitive to the length of the path between two displaced subtrees. Table S4 shows how the reconstruction accuracy as measured by the RF does not depend on the definition of distance matrix (LDN vs. LDND) neither on the specific distance-based algorithm adopted. As stressed in the main text, the use of the standard Robinson-Foulds distance can lead to a systematic larger disagreement due to the presence of non binary internal nodes in Ethnologue trees, i.e., the existence of non fully resolved subgroups of a language family. The generalized Robinson-Foulds score is not affected by this bias. The results obtained with the generalized RF score are shown in table S5. Next we consider the accuracy of the inferred trees as measured by the Quartet Distance. If we take into account the standard definition of the QD (table S6), the accuracy of the inferred trees result quite low, the average distance between the inferred and expert classification being around 45%. The adoption of the generalized Quartet Distance score allows to remove the biases due to the presence of star quartets in the classifications trees. The generalized QD scores are reported in table S7. The accuracy of the inferred trees in this case turns out to be much higher, with an average fraction of disagreement lower than 10% and with large fluctuations from a minimum of zero to a maximum of roughly 30% for the Panoan family.

2 Analysis of ASJP and ABDV databases for the Austronesian Family

We give here some supplementary information about the analysis on the Austronesian Family we presented in the main text. The set of 305 Austronesian languages taken in account is presented in Table S8, the name of languages are the ones shown in `http://language.psy.auckland.ac.nz/austronesian/`.

The Coverage of the ABVD lists for this set is of 167.32, for the ASJP is 39.83. The value of the M_{eff} is $185,23$ for the ABVD database and $46,49$ for the ASJP. The ABVD thus features higher values for both parameters.

In Table S9 we show the distance from the Ethnologue classification, as computed by all the four measures adopted (Robinson-Foulds, Generalized Robinson-Foulds, Quartet-Distance, Generalized Quartet-Distance), of the most accurate tree inferred by using the ABVD list and the most accurate tree inferred by using the ASJP list. The most accurate tree is intended to be the tree, within the ones inferred by the three considered algorithms, that features lower distance from the Ethnologue classification. The LDN definition of distance between languages, which allows for faster computations, has been used here.

All the four tree distances used, point that the inference made by using ABVD lists is more accurate than the one made by the use of ASJP lists: this is a consequence of the higher values of the Coverage and of M_{eff} featured by the ABVD database (see main text). FastSBiX appears again to be the best

Family	N	Ethn. Resolution	Coverage	M_{Eff}
Afro-Asiatic	227	0.38	43.07	39.66
Algic	28	0.22	43.93	40.34
Altaic	75	0.31	42.47	41.4
Arawakan	48	0.21	43.73	41.06
Australian	186	0.32	40.29	37.05
Austro-Asiatic	52	0.64	60.27	47.54
Austronesian	833	0.35	43.93	40.1
Border	16	0.14	33.94	32.66
Bosavi	15	0	39.33	39.72
Cariban	19	0.53	44.16	42.4
Chibchan	20	0.31	47.67	43.14
Dravidian	21	0.79	45.05	36.15
Eleman	10	0.63	44.3	41.49
Great Andamanese	10	0.25	38.3	39.91
Hmong-Mien	14	0.25	41	43.08
Hokan	24	0.57	46.22	42.29
Indo-European	210	0.36	43.69	41.16
Kadugli	11	0	41	44
Khoisan	16	0.64	41	42.67
Kiwaian	15	0	39	39.69
Lakes Plain	26	0.21	37.19	35.19
Lower Sepik-Ramu	20	0.5	31.95	28.21
Macro-Ge	24	0.32	48.5	42.72
Marind	32	0.33	34.09	30.79
Mayan	75	0.33	75.03	60.09
Mixe-Zoque	14	0.7	91	89.09
Morehead and Upper Maro Rivers	17	0.7	34	31.79
Na-Dene	22	0.6	46.45	42.42
Nakh-Daghestanian	32	0.43	41	41.29
Niger-Congo	558	0.4	41.34	39.89
Nilo-Saharan	113	0.54	42.07	40.38
Oto-Manguean	60	0.3	43.02	40.73
Panoan	18	0.31	41	42.35
Penutian	21	0.31	52.43	44.29
Quechuan	18	0.06	44.33	42.55
Salishan	12	0.4	51	45.45
Sepik	26	0.25	37.81	36.62
Sino-Tibetan	141	0.35	42.7	41.12
Sko	14	0.33	39	41.57
Tai-Kadai	56	0.31	42.11	40.78
Torricelli	31	0.21	37.06	35.63
Totonacan	14	0.25	41	44.44
Trans-New Guinea	293	0.31	40.02	36.01
Tucanoan	14	0.59	47.32	42.92
Tupian	47	0.24	44.75	41.09
Uralic	23	0.43	48.74	42.71
Uto-Aztecan	81	0.34	45.5	41.37
West Papuan	34	0.22	38.21	36.17
Western Fly	39	0	37.77	37.01

Table S1. Statistical Properties of the families in the ASJP database. In this table we report the number N of languages and the resolution of the Ethnologue classification for each family in the ASJP data set. We also show two statistical properties of the lists of words: the M_{eff} of words and the Coverage. See the text for the definitions.

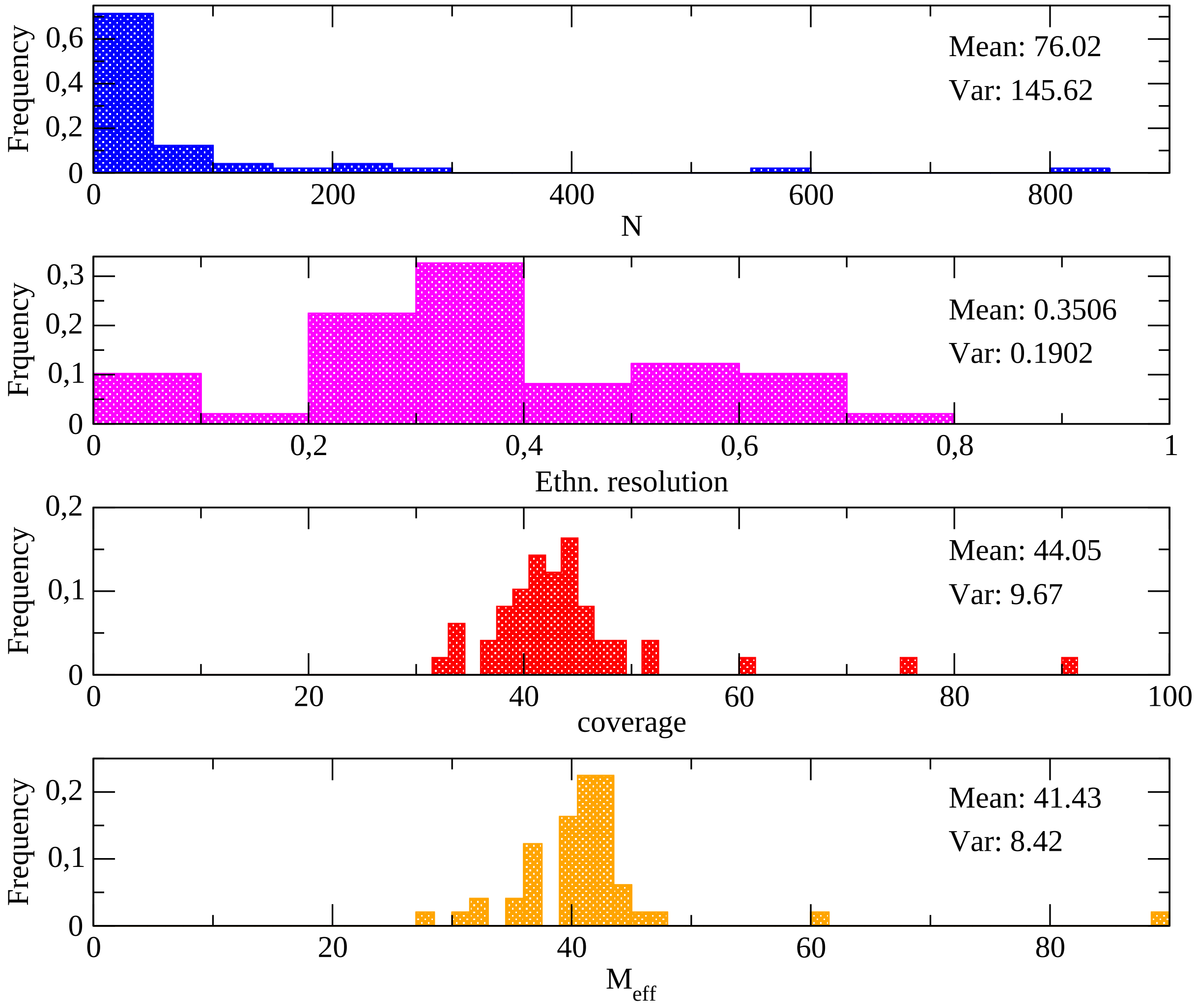

Figure S1. Histograms of ASJP and Ethnologue features. Histograms of the number N of languages in each family, the Ethnologue resolution for each family, M_{eff} and the Coverage of the words lists in each family are reported. While both N and the Ethnologue resolution feature a great variability across the world family, M_{eff} and the Coverage are almost constant in the lists of words related to language families in the ASJP database.

	N	Ethn. resolution	Coverage	M_{eff}
**RF** - FastME	0.3514	-0.1298	-0.1542	-0.1903
**RF** - NJ	0.3097	-0.1459	-0.1654	-0.2228
**RF** - FastSBiX	0.3488	-0.2108	-0.1045	-0.1283
**QD** - FastME	-0.1458	-0.6704	-0.2605	-0.1066
**QD** - NJ	-0.1847	-0.6901	-0.2623	-0.1120
**QD** - FastSBiX	-0.211	-0.6717	-0.2494	-0.0926
**GRF** - FastME	0.1925	0.3414	0.0453	0.0024
**GRF** - NJ	0.1829	0.3950	0.0833	0.0512
**GRF** - FastSBiX	0.1869	0.3543	0.1230	0.0985
**GQD** - FastME	0.0686	0.1079	-0.0923	-0.0749
**GQD** - NJ	0.1338	0.1352	-0.0855	-0.0599
**GQD** - FastSBiX	0.0084	0.1392	-0.0597	-0.0256

Table S2. Pearson's r between reconstruction accuracy and parameters of ASJP and Ethnologue databases. This table shows the Pearson's r between the accuracy (as measured by the RF, the QD, the GRF and the GDQ) of the trees inferred with FastSBiX, FastME and Neighbour-Joining, starting from the LDND matrices, and different properties of the lists of words of each family in the ASJP database. In particular, we compute the Pearson's r between the estimated accuracy of the inferred trees and the number N of languages in a family (first column), the Ethnologue Resolution (second column), the Coverage of words in lists (third column) and the effective length of the word-lists M_{eff} (last column).

distance-based algorithm for tree reconstruction, both considering the GRF and GQD distances.

3 World maps

We report here world maps showing the Generalized Quartet Distance between inferred trees and Ethnologue classifications. Trees inferred starting from LDN matrices are shown in Fig. S2 (with Neighbour-Joining), Fig. S3 (FastME) and in Fig. S4. Trees inferred starting from LDND matrices are shown in Fig. S5 (Neighbour-Joining), Fig. S3 (FastME). In the main text we have shown the map of the LDND-inferred trees with FastSBiX. We recall that the colors code, for each family f, the following quantity $X_f = 2 * GQD(f)/GQD_{random}(f)$, where $GQD_{random}(f)$ represents the value of the GQD obtained as average over 10 random trees with the same number of leaves (languages) of the family f (see main text). X_f quantifies the level of accuracy of the reconstruction with respect to a null model. Blue families are those for which the accuracy is very high while for red families the accuracy is very low, i.e., smaller than half the the random value. Yellow regions on the maps are related to (non-significant) families with null Ethnologue resolution, for which a random reconstruction would get a null value of the GQD.

The difference of the accuracy of the different algorithms is more evident in some regions such as the whole Africa, the Oceania and the east-Europe, whereas areas such as the whole America do not exhibit big differences in all the maps. This visual analysis immediately reveals the main conclusions we have drawn in the main text. Recalling that darker colors (i.e., color with a higher percentage of blue) point to a better accuracy, we see that the sensible regions always get darker while going from LDN maps to LDND ones. This behaviour reveals a slightly better accuracy achieved with the former definition of distance between lists of words. The effect of the different distance-based algorithms used for the reconstruction is visibly more evident. Neighbour-Joining maps displays more red regions than FastME maps; these sensible regions always get darker when observing FastSBiX maps. This visual analysis,

	N	Ethn. resolution	Coverage	M_{eff}
**RF** - FastME	0.3593	-0.1370	-0.1497	-0.1837
**RF** - NJ	0.3155	-0.1651	-0.1624	-0.2171
**RF** - FastSBiX	0.3604	-0.2213	0.0959	-0.1203
**QD** - FastME	-0.0715	-0.4126	-0.2459	-0.1231
**QD** - NJ	-0.1309	-0.4476	-0.2499	-0.1322
**QD** - FastSBiX	-0.1713	-0.4132	-0.1022	-0.1023
**GRF** - FastME	0.1542	0.1403	-0.0078	-0.0120
**GRF** - NJ	0.1445	0.2216	0.0355	0.0415
**GRF** - FastSBiX	0.1482	0.1591	0.0780	0.0931
**GQD** - FastME	0.0285	-0.1111	-0.1433	-0.0907
**GQD** - NJ	0.0994	-0.0636	-0.1334	-0.0740
**GQD** - FastSBiX	-0.0321	-0.0511	0.0883	-0.0374
$\mathbf{GQD/GQD}_{\mathrm{random}}$ - FastME	0.1127	-0.1192	-0.1507	-0.0990
$\mathbf{GQD/GQD}_{\mathrm{random}}$ - NJ	0.1969	-0.0724	-0.1394	-0.0821
$\mathbf{GQD/GQD}_{\mathrm{random}}$ - FastSBiX	0.0348	-0.0576	0.0746	-0.0445
$\mathbf{GRF/GRF}_{\mathrm{random}}$ - FastME	0.1157	0.0779	-0.0391	-0.0229
$\mathbf{GRF/GRF}_{\mathrm{random}}$ - NJ	0.1084	0.1610	0.0026	0.0276
$\mathbf{GRF/GRF}_{\mathrm{random}}$ - FastSBiX	0.1097	0.0994	0.0434	0.0782

Table S3. Pearson's r between reconstruction accuracy and parameters of ASJP and Ethnologue databases. We show here the same quantities as in table S2, but the correlations are now computed without taking in account families in which the Ethnologue database does not provide a classification (it results in a star tree). Here, the measures $GQD/GQD_{\mathrm{random}}$ and $GRF/GRF_{\mathrm{random}}$ are also taken into account. They allow for an analysis of the correlations of the accuracy of the inference with respect to a null model. Correlations are still very low for all the considered quantities, FastSBiX revealing for all of them the lowest correlation.

thus, enlightens once again the suitability of the noise-reduction procedure used by FastSBiX to infer the correct topology of language trees.

We finally list all the regions where we included the average statistics of more than one language family: In the Nilo-Saharan region we included Kadugli and Nilo-Saharan; in the Papuan-labelled region we included Bosavi, Eleman, Kiwaian, Sko, Western Fly, Marind, Sepik, West Papuan, Trans-New Guinea, Torricelli, Morehead and Upper Maro Rivers, Lakes Plain, Border, Lower Sepik-Ramu.

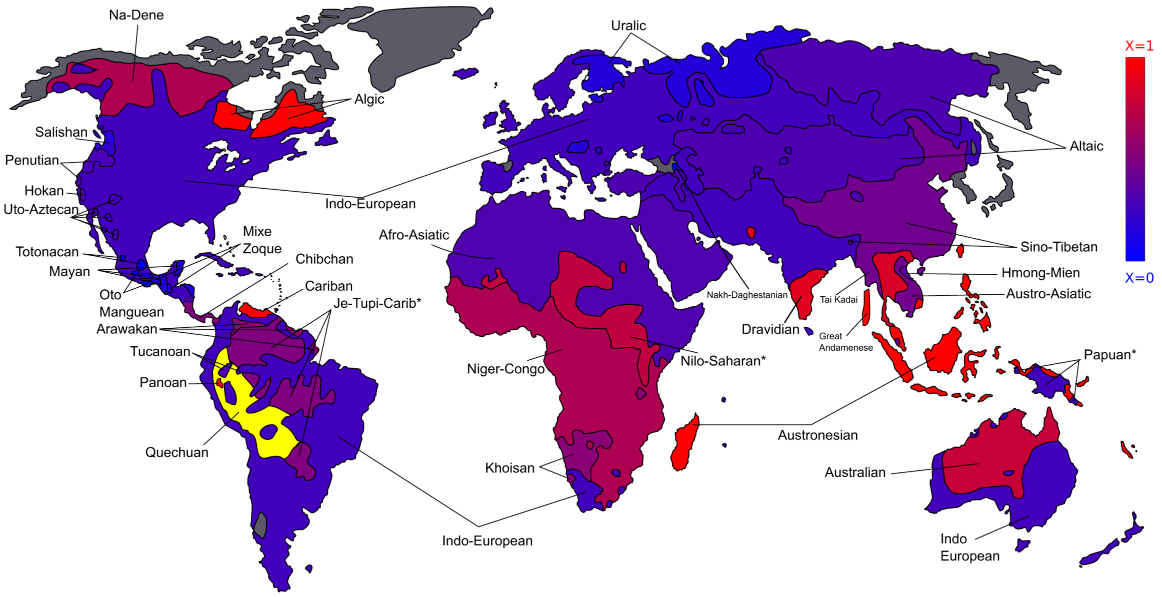

Figure S2. Accuracy of the reconstruction across the planet. NJ, LDN matrix. This map represents the level of accuracy of the Neighbour-Joining algorithm on several language families throughout the world. Trees Inferred with LDN matrices. The colors code the values of the Generalized Quartet Distance (GQD) between the trees inferred with the NJ algorithm and the LDN definition of distance for each language family included in the ASJP database and the corresponding Ethnologue classifications. The GQD is normalized with the corresponding random value (see text for details). On the one hand blue regions corresponds to language families for which the inferred trees strongly agree with the Ethnologue classification. On the other hand red regions corresponds to poorly reconstructed language families. Yellow is for the families in which a random reconstruction would get a GQD score of zero, meaning that the Ethnologue classification has a null resolution (the corresponding tree is a star). Grey areas are those for which no data are present in the databases adopted for the reconstruction. Asterisks are for regions which include more than one family of languages.

ROBINSON-FOULDS DISTANCE

	LDN			LDND			
	**Neighbour-Joining**	**FastME**	**FastSBiX**	**Neighbour-Joining**	**FastME**	**FastSBiX**	**RANDOM**
**AFRICA**							
Khoisan	**0.4688**	**0.4688**	**0.4688**	**0.4688**	**0.4688**	**0.4688**	0.9905
Niger-Congo	0.4964	0.4946	**0.4910**	0.5000	0.5018	0.4964	0.9995
Nilo-Saharan	0.4292	0.4292	0.4027	0.4204	0.4027	**0.3938**	0.9952
Kadugli	**0.3636**	**0.3636**	**0.3636**	**0.3636**	**0.3636**	**0.3636**	1.0000
Afro-Asiatic	0.4626	0.4537	0.4537	0.4626	**0.4449**	**0.4449**	0.9994
**EURASIA**							
Indo-European	0.5500	**0.5310**	0.5405	0.5500	0.5357	0.5405	1.0000
Uralic	0.3696	0.3696	0.3696	0.3696	0.3696	**0.3261**	0.9862
Altaic	**0.5400**	**0.5400**	**0.5400**	**0.5400**	**0.5400**	0.5533	0.9979
Dravidian	0.5000	0.5000	**0.3571**	0.4524	0.4524	**0.3571**	0.9879
Nakh-Daghestanian	**0.2813**	**0.2813**	**0.2813**	**0.2813**	**0.2813**	**0.2813**	1.0000
Sino-Tibetan	0.5390	0.5248	**0.5177**	0.5390	**0.5177**	**0.5177**	0.9989
Hmong-Mien	**0.3571**	**0.3571**	**0.3571**	**0.3571**	**0.3571**	**0.3571**	1.0000
Tai-Kadai	**0.4911**	**0.4911**	**0.4911**	**0.4911**	**0.4911**	**0.4911**	1.0000
Great Andamanese	**0.4000**	**0.4000**	**0.4000**	**0.4000**	**0.4000**	**0.4000**	1.0000
Austro-Asiatic	0.3462	0.3462	0.3654	**0.3269**	0.3462	0.3654	0.9975
**PACIFIC**							
Austronesian	0.5258	0.5563	0.5258	**0.5246**	0.5330	**0.5246**	0.9994
Border	0.4375	0.4375	**0.3750**	0.4375	0.4375	**0.3750**	1.0000
Bosavi	**0.4000**	**0.4000**	**0.4000**	**0.4000**	**0.4000**	**0.4000**	1.0000
Kiwaian	**0.4000**	**0.4000**	**0.4000**	**0.4000**	**0.4000**	**0.4000**	1.0000
Eleman	**0.1000**	**0.1000**	**0.1000**	**0.1000**	**0.1000**	**0.1000**	0.9333
Lower Sepik-Ramu	**0.2000**	0.2500	**0.2000**	0.2500	0.2500	**0.2000**	0.9923
Lakes Plain	**0.4231**	**0.4231**	**0.4231**	**0.4231**	**0.4231**	**0.4231**	0.9929
Marind	**0.3438**	**0.3438**	**0.3438**	**0.3438**	**0.3438**	**0.3438**	0.9947
Morehead and Upper Maro Rivers	**0.3235**	**0.3235**	**0.3235**	**0.3235**	**0.3235**	**0.3235**	1.0000
Sepik	0.4038	0.3654	0.3654	0.3654	**0.3269**	0.3654	1.0000
Sko	**0.1429**	**0.1429**	**0.1429**	**0.1429**	**0.1429**	**0.1429**	0.9667
Australian	**0.4113**	0.4274	0.4220	**0.4113**	0.4274	0.4167	1.0000
Torricelli	0.4839	0.4839	0.4839	**0.4516**	**0.4516**	0.4839	1.0000
Trans-New Guinea	0.4454	**0.4386**	0.4420	**0.4386**	**0.4386**	**0.4386**	0.9995
Western Fly	**0.4615**	**0.4615**	**0.4615**	**0.4615**	**0.4615**	**0.4615**	1.0000
West Papuan	0.4706	0.4412	**0.4118**	0.4706	0.4412	0.4412	1.0000
**AMERICA**							
Na-Dene	0.5455	0.5000	**0.4545**	0.5455	0.5000	**0.4545**	0.9933
Uto-Aztecan	0.1914	0.1914	0.1914	0.1914	**0.1790**	0.1914	0.9959
Algic	0.5000	0.5000	0.5000	**0.4643**	0.5000	0.5000	1.0000
Panoan	**0.5000**	**0.5000**	**0.5000**	**0.5000**	**0.5000**	**0.5000**	1.0000
Salishan	**0.2917**	**0.2917**	**0.2917**	**0.2917**	**0.2917**	**0.2917**	1.0000
Quechuan	**0.4167**	**0.4167**	**0.4167**	**0.4167**	**0.4167**	**0.4167**	1.0000
Penutian	0.2619	**0.2143**	**0.2143**	0.2619	**0.2143**	**0.2143**	0.9931
Tupian	**0.4681**	**0.4681**	**0.4681**	**0.4681**	**0.4681**	**0.4681**	1.0000
Hokan	0.4375	**0.3958**	**0.3958**	**0.3958**	0.4375	**0.3958**	1.0000
Macro-Ge	**0.4583**	**0.4583**	0.5000	0.5000	**0.4583**	0.5000	1.0000
Oto-Manguean	**0.3583**	**0.3583**	**0.3583**	**0.3583**	**0.3583**	**0.3583**	0.9945
Tucanoan	0.4286	0.4286	0.4286	**0.3571**	0.4286	0.4286	0.9923
Arawakan	0.4375	0.4375	0.4375	0.4375	0.4375	**0.4167**	1.0000
Cariban	0.5263	0.5263	**0.4737**	0.5263	0.5789	0.5263	0.9833
Mixe-Zoque	**0.1786**	0.2500	0.2500	**0.1786**	0.2500	0.2500	0.9867
Mayan	0.4600	**0.4067**	0.4333	0.4467	**0.4067**	0.4333	1.0000
Chibchan	**0.3500**	**0.3500**	**0.3500**	0.4000	0.4000	0.4000	0.9900
Totonacan	**0.2143**	**0.2143**	**0.2143**	**0.2143**	**0.2143**	**0.2143**	1.0000
AVERAGE	0.3998	0.3970	**0.3898**	0.3963	0.3962	0.3910	0.9951

Table S4. Robinson-Foulds distance between inferred trees and the Ethnologue classification. This table shows the Robinson-Foulds distances between the inferred trees and the Ethnologue classification for each language family in the ASJP database. For each language family we report the RF distance obtained using both the LDN and the LDND distances between languages in the framework of the NJ, FastMe and FastSBiX algorithms. Finally the last column reports the results obtained in the comparison of the Ethnologue classifications with specific artificial random trees for each language family. First of all the RF distances for the actual inferred trees are much smaller than the corresponding random case. Still these values are always very high independently on the matrix distance used and of the specific phylogenetic algorithm adopted. A systematic effect that increases the errors is the bias induced by the non-binary nodes in the Ethnologue trees.

GENERALIZED ROBINSON-FOULDS SCORE

	LDN			LDND			
	**Neighbour-Joining**	**FastME**	**FastSBiX**	**Neighbour-Joining**	**FastME**	**FastSBiX**	**RANDOM**
			**AFRICA**				
Khoisan	**0.4615**	**0.4615**	**0.4615**	**0.4615**	**0.4615**	**0.4615**	0.9769
Niger-Congo	0.4127	**0.3891**	0.4091	0.4109	0.4018	0.4127	0.9911
Nilo-Saharan	0.3119	0.3395	0.2844	0.3028	0.2844	**0.2752**	0.9853
Kadugli	**0.0000**	**0.0000**	**0.0000**	**0.0000**	**0.0000**	**0.0000**	**0.0000**
Afro-Asiatic	0.2500	0.2325	0.2193	0.2544	0.2237	**0.2149**	0.9908
			**EURASIA**				
Indo-European	0.3505	0.3411	0.3224	0.3411	0.3271	**0.3084**	0.9785
Uralic	**0.1500**	**0.1500**	**0.1500**	**0.1500**	**0.1500**	**0.1500**	0.9450
Altaic	**0.3108**	**0.3108**	0.3243	**0.3108**	**0.3108**	0.3378	0.9527
Dravidian	0.6111	0.6111	0.4444	0.5556	0.5556	**0.3889**	0.9889
Nakh-Daghestanian	**0.0690**	**0.0690**	**0.0690**	**0.0690**	**0.0690**	0.1034	0.9655
Sino-Tibetan	0.4348	0.4275	**0.4130**	0.4348	**0.4130**	**0.4130**	0.9841
Hmong-Mien	**0.1818**	**0.1818**	0.2727	0.2727	**0.1818**	0.2727	0.8636
Tai-Kadai	0.4118	**0.3922**	0.4118	0.4118	**0.3922**	0.4118	0.9725
Great Andamanese	**0.2857**	**0.2857**	**0.2857**	**0.2857**	**0.2857**	**0.2857**	0.4143
Austro-Asiatic	0.3469	0.3469	0.3061	**0.2245**	0.2449	0.3265	0.9980
			**PACIFIC**				
Austronesian	0.3881	0.4063	0.3820	0.3844	0.3942	**0.3723**	0.9793
Border	0.0769	0.0769	**0.0000**	0.0769	0.0769	**0.0000**	0.7538
Bosavi	**0.0000**	**0.0000**	**0.0000**	**0.0000**	**0.0000**	**0.0000**	**0.0000**
Kiwaian	**0.0000**	**0.0000**	**0.0000**	**0.0000**	**0.0000**	**0.0000**	**0.0000**
Eleman	**0.0000**	**0.0000**	**0.0000**	**0.0000**	**0.0000**	**0.0000**	0.8429
Lower Sepik-Ramu	**0.0000**	0.1176	**0.0000**	0.0588	0.0588	**0.0000**	0.9765
Lakes Plain	**0.1739**	**0.1739**	**0.1739**	**0.1739**	**0.1739**	**0.1739**	0.9043
Marind	**0.0690**	**0.0690**	**0.0690**	**0.0690**	**0.0690**	**0.0690**	0.9517
Morehead and Upper Maro Rivers	0.1429	0.1429	0.1429	**0.0714**	0.1429	**0.0714**	0.9500
Sepik	0.0870	0.0435	0.0435	0.0435	**0.0000**	0.0435	0.9391
Sko	**0.0000**	**0.0000**	**0.0000**	**0.0000**	**0.0000**	**0.0000**	0.5600
Australian	**0.3653**	0.4012	0.3772	0.3832	0.3892	**0.3653**	0.9934
Torricelli	0.2143	0.2500	0.2143	0.2143	**0.1786**	0.2500	0.9000
Trans-New Guinea	0.2544	0.2230	0.2474	**0.2195**	0.2404	0.2265	0.9868
Western Fly	**0.0000**	**0.0000**	**0.0000**	**0.0000**	**0.0000**	**0.0000**	**0.0000**
West Papuan	0.1935	0.1290	**0.0968**	0.2258	0.1613	0.1613	0.9129
			**AMERICA**				
Na-Dene	0.7368	0.6316	**0.5789**	0.7368	0.6316	**0.5789**	0.9842
Uto-Aztecan	0.1622	0.1622	0.1622	0.1622	**0.1351**	0.1892	0.9405
Algic	0.3846	0.3846	**0.3462**	0.3846	0.3846	**0.3462**	0.9385
Panoan	0.8000	0.8000	**0.7333**	0.8000	0.8000	**0.7333**	0.9800
Salishan	**0.1111**	**0.1111**	**0.1111**	**0.1111**	**0.1111**	**0.1111**	0.9556
Quechuan	**0.0000**	**0.0000**	**0.0000**	**0.0000**	**0.0000**	**0.0000**	**0.0000**
Penutian	0.1667	**0.0556**	**0.0556**	0.1111	**0.0556**	**0.0556**	0.9667
Tupian	0.4444	0.4000	0.4222	**0.3556**	0.4667	0.4667	0.9867
Hokan	0.4000	**0.3500**	**0.3500**	**0.3500**	0.4500	0.4000	0.9800
Macro-Ge	0.3810	0.3810	**0.3333**	0.3810	**0.3333**	0.3810	0.9333
Oto-Manguean	**0.0357**	**0.0357**	**0.0357**	**0.0357**	**0.0357**	**0.0357**	0.9393
Tucanoan	0.1875	0.1875	0.2500	**0.1250**	0.1875	0.1875	0.9625
Arawakan	0.2195	0.1951	0.1951	0.2195	0.1951	**0.1707**	0.9463
Cariban	0.8125	**0.7500**	**0.7500**	**0.7500**	0.8750	0.8750	0.9500
Mixe-Zoque	**0.1111**	0.2222	0.2222	**0.1111**	0.2222	0.2222	0.9889
Mayan	0.1972	**0.1268**	0.1549	0.1831	**0.1268**	0.1549	0.9394
Chibchan	0.4000	0.4000	**0.3333**	0.5333	0.5333	0.5333	0.9600
Totonacan	**0.0000**	**0.0000**	**0.0000**	**0.0000**	**0.0000**	**0.0000**	0.7571
AVERAGE	0.2470	0.2401	**0.2276**	0.2399	0.2394	0.2354	0.8320

Table S5. Generalized Robinson-Foulds scores between inferred trees and the Ethnologue classification. This table shows the generalized Robinson-Foulds scores between the inferred trees and the Ethnologue classification. The legend is the same as in Table S4. The generalization has been introduced to get rid of biases in the Robinson-Foulds distance, due to the presence of non binary nodes in the classifications trees. The average scores are now much smaller (roughly for a factor 2) than for the usual Robinson-Foulds distance.

QUARTET DISTANCE

	LDN			LDND			
	**Neighbour-Joining**	**FastME**	**FastSBiX**	**Neighbour-Joining**	**FastME**	**FastSBiX**	**RANDOM**
**AFRICA**							
Khoisan	**0.2885**	**0.2885**	0.2984	**0.2885**	**0.2885**	0.2984	0.7088
Niger-Congo	0.3396	0.5057	0.3401	0.3471	0.3355	**0.2853**	0.6120
Nilo-Saharan	0.3420	0.3484	0.3311	0.2141	0.2214	**0.2128**	0.6912
Kadugli	**1.0000**	**1.0000**	**1.0000**	**1.0000**	**1.0000**	**1.0000**	**1.0000**
Afro-Asiatic	0.2550	0.2808	0.2556	0.2508	0.2371	**0.2083**	0.6440
**EURASIA**							
Indo-European	0.1694	0.1743	0.1661	0.1650	0.1632	**0.1626**	0.6065
Uralic	0.5007	**0.4949**	**0.4949**	0.5007	**0.4949**	0.4963	0.8087
Altaic	0.2800	0.2813	**0.2733**	0.2800	0.2813	0.2891	0.7167
Dravidian	0.3666	0.3666	0.3793	0.3101	0.3078	**0.2526**	0.7095
Nakh-Daghestanian	**0.2613**	**0.2613**	**0.2613**	**0.2613**	**0.2613**	0.2969	0.7455
Sino-Tibetan	0.5051	0.4829	0.4828	0.4900	0.4907	**0.4821**	0.7879
Hmong-Mien	**0.4016**	**0.4016**	0.4066	0.4066	**0.4016**	0.4066	0.7690
Tai-Kadai	0.3772	0.3523	0.3300	0.3701	0.3523	**0.3245**	0.7093
Great Andamanese	**0.9571**	**0.9571**	**0.9571**	**0.9571**	**0.9571**	**0.9571**	0.9805
Austro-Asiatic	0.3336	0.2923	0.2593	**0.2549**	0.2558	0.2763	0.7381
**PACIFIC**							
Austronesian	0.3731	0.3721	0.3306	0.2963	0.3976	**0.2746**	0.6650
Border	0.6269	0.6269	**0.5692**	0.6269	0.6269	**0.5692**	0.8344
Bosavi	**1.0000**	**1.0000**	**1.0000**	**1.0000**	**1.0000**	**1.0000**	**1.0000**
Kiwaian	**1.0000**	**1.0000**	**1.0000**	**1.0000**	**1.0000**	**1.0000**	**1.0000**
Eleman	**0.1190**	**0.1190**	**0.1190**	**0.1190**	**0.1190**	**0.1190**	0.6895
Lower Sepik-Ramu	**0.1969**	0.2140	**0.1969**	0.2004	0.2004	**0.1969**	0.7354
Lakes Plain	**0.4813**	**0.4813**	**0.4813**	**0.4813**	**0.4813**	**0.4813**	0.7817
Marind	**0.2000**	**0.2000**	**0.2000**	**0.2000**	**0.2000**	**0.2000**	0.7345
Morehead and Upper Maro Rivers	**0.2664**	0.3496	**0.2664**	0.3345	0.3546	0.3345	0.7282
Sepik	0.3466	0.3523	0.3145	0.3451	**0.3130**	0.3451	0.7738
Sko	**0.4857**	**0.4857**	**0.4857**	**0.4857**	**0.4857**	**0.4857**	0.7571
Australian	0.5674	**0.5553**	0.5685	0.5792	0.5666	0.5573	0.7824
Torricelli	0.4898	0.4625	0.4625	0.4513	**0.4493**	0.4625	0.8037
Trans-New Guinea	0.4593	0.4592	0.4527	0.4532	**0.4474**	0.4505	0.7265
Western Fly	**1.0000**	**1.0000**	**1.0000**	**1.0000**	**1.0000**	**1.0000**	**1.0000**
West Papuan	0.2529	0.2455	**0.2209**	0.2471	0.2397	0.2397	0.7309
**AMERICA**							
Na-Dene	0.3794	0.3753	**0.3671**	0.3794	0.3753	**0.3671**	0.7234
Uto-Aztecan	**0.2914**	**0.2914**	**0.2914**	**0.2914**	0.2945	0.2965	0.7419
Algic	**0.5673**	**0.5673**	0.5695	0.5885	0.5941	0.5782	0.7717
Panoan	0.6876	0.6909	**0.6791**	0.6928	0.6909	0.6827	0.8152
Salishan	**0.3354**	**0.3354**	**0.3354**	**0.3354**	**0.3354**	**0.3354**	0.7578
Quechuan	**1.0000**	**1.0000**	**1.0000**	**1.0000**	**1.0000**	**1.0000**	**1.0000**
Penutian	0.2145	**0.1370**	**0.1370**	0.2092	**0.1370**	**0.1370**	0.7068
Tupian	0.5359	0.5188	0.5109	**0.5055**	0.5135	0.5209	0.8169
Hokan	0.2577	0.2582	0.2690	**0.2447**	0.2733	0.2654	0.7255
Macro-Ge	**0.4343**	0.4551	0.4650	0.4789	0.4645	0.4823	0.7764
Oto-Manguean	**0.3767**	**0.3767**	**0.3767**	**0.3767**	**0.3767**	**0.3767**	0.7855
Tucanoan	0.2608	0.2608	0.3351	**0.2567**	0.2608	0.2608	0.7166
Arawakan	0.3494	0.3685	0.3212	0.3541	0.3685	**0.3133**	0.7363
Cariban	0.6370	0.5986	0.5929	**0.5815**	0.6391	0.6373	0.7304
Mixe-Zoque	**0.2384**	0.2566	0.2566	**0.2384**	0.2566	0.2566	0.7402
Mayan	0.2060	**0.1925**	0.1943	0.2057	**0.1925**	0.1943	0.7074
Chibchan	**0.5788**	**0.5788**	0.6023	0.6199	0.6199	0.6199	0.8291
Totonacan	**0.7000**	**0.7000**	**0.7000**	**0.7000**	**0.7000**	**0.7000**	0.9224
AVERAGE	0.4550	0.4566	0.4471	0.4485	0.4494	**0.4426**	0.7744

Table S6. Quartet Distance between inferred trees and the Ethnologue classification. This table shows the Quartet Distance between the inferred trees and the Ethnologue classification for each language family in the ASJP database. The legend is the same as in Table S4. In this case the scores are affected by the presence of star quartets of taxa in the Ethnologue trees, as a consequence of the existence of non-binary nodes.

GENERALIZED QUARTET DISTANCE

	LDN			LDND			
	**Neighbour-Joining**	**FastME**	**FastSBiX**	**Neighbour-Joining**	**FastME**	**FastSBiX**	**RANDOM**
**AFRICA**							
Khoisan	**0.1809**	**0.1809**	0.1923	**0.1809**	**0.1809**	0.1923	0.6650
Niger-Congo	0.1756	0.3830	0.1763	0.1850	0.1705	**0.1078**	0.5158
Nilo-Saharan	0.2436	0.2510	0.2310	0.0966	0.1051	**0.0951**	0.6451
Kadugli	**0.0000**	**0.0000**	**0.0000**	**0.0000**	**0.0000**	**0.0000**	**0.0000**
Afro-Asiatic	0.0895	0.1209	0.0901	0.0843	0.0676	**0.0323**	0.5647
**EURASIA**							
Indo-European	0.0738	0.0793	0.0701	0.0689	0.0669	**0.0662**	0.5609
Uralic	0.0458	**0.0347**	**0.0347**	0.0458	**0.0347**	0.0373	0.6355
Altaic	0.0990	0.1006	**0.0906**	0.0988	0.1006	0.1102	0.6451
Dravidian	0.2978	0.2978	0.3119	0.2352	0.2327	**0.1715**	0.6779
Nakh-Daghestanian	**0.0179**	**0.0179**	**0.0179**	**0.0179**	**0.0179**	0.0653	0.6632
Sino-Tibetan	0.1411	0.1026	0.1024	0.1149	0.1161	**0.1012**	0.6319
Hmong-Mien	**0.1243**	**0.1243**	0.1316	0.1316	**0.1243**	0.1316	0.6620
Tai-Kadai	0.2838	0.2553	0.2296	0.2758	0.2553	**0.2232**	0.6659
Great Andamanese	0.6786	0.6786	0.6786	0.6786	0.6786	0.6786	**0.6283**
Austro-Asiatic	0.1489	0.0962	0.0542	**0.0485**	0.0496	0.0757	0.6658
**PACIFIC**							
Austronesian	0.3006	0.2995	0.2523	0.2149	0.3279	**0.1907**	0.5132
Border	0.1339	0.1339	**0.0000**	0.1339	0.1339	**0.0000**	0.6156
Bosavi	**0.0000**	**0.0000**	**0.0000**	**0.0000**	**0.0000**	**0.0000**	**0.0000**
Kiwaian	**0.0000**	**0.0000**	**0.0000**	**0.0000**	**0.0000**	**0.0000**	**0.0000**
Eleman	**0.0000**	**0.0000**	**0.0000**	**0.0000**	**0.0000**	**0.0000**	0.5705
Lower Sepik-Ramu	**0.0000**	0.0213	**0.0000**	0.0044	0.0044	**0.0000**	0.6717
Lakes Plain	**0.2096**	**0.2096**	**0.2096**	**0.2096**	**0.2096**	**0.2096**	0.6675
Marind	**0.0284**	**0.0284**	**0.0284**	**0.0284**	**0.0284**	**0.0284**	0.6763
Morehead and Upper Maro Rivers	**0.0673**	0.1732	**0.0673**	0.1540	0.1797	0.1540	0.6549
Sepik	0.0490	0.0573	0.0022	0.0468	**0.0000**	0.0468	0.6716
Sko	**0.0000**	**0.0000**	**0.0000**	**0.0000**	**0.0000**	**0.0000**	0.2714
Australian	0.2405	**0.2195**	0.2426	0.2614	0.2391	0.2230	0.6182
Torricelli	0.1592	0.1144	0.1144	0.0959	**0.0926**	0.1144	0.6769
Trans-New Guinea	0.1221	0.1221	0.1114	0.1122	**0.1029**	0.1078	0.5561
Western Fly	**0.0000**	**0.0000**	**0.0000**	**0.0000**	**0.0000**	**0.0000**	**0.0000**
West Papuan	0.0714	0.0623	**0.0317**	0.0642	0.0550	0.0550	0.6654
**AMERICA**							
Na-Dene	0.2251	0.2200	**0.2098**	0.2251	0.2200	**0.2098**	0.6548
Uto-Aztecan	**0.0911**	**0.0911**	**0.0911**	**0.0911**	0.0951	0.0976	0.6686
Algic	**0.3609**	**0.3609**	0.3641	0.3920	0.4003	0.3768	0.6628
Panoan	0.4885	0.4937	**0.4746**	0.4970	0.4937	0.4805	0.6974
Salishan	**0.0600**	**0.0600**	**0.0600**	**0.0600**	**0.0600**	**0.0600**	0.6577
Quechuan	**0.0000**	**0.0000**	**0.0000**	**0.0000**	**0.0000**	**0.0000**	**0.0000**
Penutian	0.0958	**0.0066**	**0.0066**	0.0897	**0.0066**	**0.0066**	0.6626
Tupian	0.1783	0.1478	0.1340	**0.1244**	0.1386	0.1517	0.6757
Hokan	0.1006	0.1012	0.1143	**0.0848**	0.1195	0.1099	0.6674
Macro-Ge	**0.1598**	0.1907	0.2052	0.2260	0.2046	0.2310	0.6677
Oto-Manguean	**0.0015**	**0.0015**	**0.0015**	**0.0015**	**0.0015**	**0.0015**	0.6857
Tucanoan	0.1105	0.1105	0.2001	**0.1056**	0.1105	0.1105	0.6593
Arawakan	0.1859	0.2098	0.1506	0.1916	0.2098	**0.1407**	0.6702
Cariban	0.5455	0.4973	0.4902	**0.4761**	0.5480	0.5458	0.6625
Mixe-Zoque	**0.0528**	0.0754	0.0754	**0.0528**	0.0754	0.0754	0.6772
Mayan	0.0469	**0.0307**	0.0327	0.0465	**0.0307**	0.0327	0.6483
Chibchan	**0.1673**	**0.1673**	0.2140	0.2488	0.2488	0.2488	0.6625
Totonacan	**0.0000**	**0.0000**	**0.0000**	**0.0000**	**0.0000**	**0.0000**	0.2224
AVERAGE	0.0984	0.0965	0.0870	0.0884	0.0894	**0.0825**	0.4122

Table S7. Generalized Quartet Distance between inferred trees and the Ethnologue classification. The generalized QD, shown in this table, quantifies the overall disagreement between inferred trees and the Ethnologue classifications. The legend is the same as in Table S4. The errors are now extremely low being always lower than 10%. This means that distance based approaches lead to accurate and robust classifications of the languages taken in account.

Acehnese	Gapapaiwa	Kwara'ae (Solomon Islands')	Muyuw	Solos
Aklanon-Bisayan	Gaddang	Lahanan	Nalik	Soboyo
Alune	Gayo	Lala	Nanggu	Sowa
Amahai	Gedaged	Lamalera (lembata)	Nauna	Suau
Amara	Geser	Lamboya	Nehan	Sudest
Ambai (Yapen)	Ghari	Lamogai (Mulakaino)	Nengone	Surigaonon
Anakalang	Gorontalo (Hulondalo)	Lampung	Nggao (Poro)	Tabar
Apma Suru Kavian	Gumawana	Langalanga	Nggela	Tagabili
Aputai	Haku	Lau	Nila	Tagalog
Araki (Southwest Santo)	Hawaiian	Leipon	Niue	Tagbanwa, Aborlan Dialect
Arosi (Tawatana Village)	Hiligaynon	Lengo	Nukuoro	Tagbanwa, Kalamian, Coron Island Dialect
As	Hitu (Ambon)	Letinese	Numfor	Tahitian (Modern)
Asumboa	Hiw	Levei	Ogan	Taiof
Bali	Hoava	Likum	Oroha	Takia
Banggai (W.dialect)	Iaai	Lio, Flores Tongah	Paiwan	Talur
Banoni	Iban	Longgu	Palauan	Tanga
Bantik	Ibanag	Loniu	Palu'e (Nitung)	Tarpia
Baree	Idaan	Lou	Pangasinan	Tausug, Jolo Dialect
Barok	Iliun	Luang	Papora	Tawala
Bauro (Baroo Village)	Ilokano	Luangiua	Patpatar	Teanu
Belait	Ilongot;Kakiduge:n	Lunga Lunga (Minigir)	Paulohi	Tela-Masbuar
Besemah	Imorod	Lungga	Pazeh	Teop
Biga (Misool)	Imroing	Maanyan	Penrhyn	Teun
Bilur	Inabaknon	Madak	Perai	Thao
Bima	Indonesian	Madurese	Phan Rang Cham (Eastern Cham)	Tiang
Bintulu	Inibaloi	Magori (South East Papua)	Pukapuka	Tigak
Binukid	Iranun	Maisin	Pulo-Annan	Tikopia
Blablanga	Itneg, Binongan	Malango	Puluwatese	Timugon (Murut)
Bobot	Ivatan, Basco Dialect	Maleu	Puyuma	Tolo
Bolaang Mongondow	Jawe	Mamanwa	Raga	Tongan
Bonerate	Kadorih	Mamboru	Rarotongan	Tonsea
Bonfia	Kahua	Manam	Rejang Rejang	Tontemboan
Bughotu	Kaidipang	Manggarai	Rennellese	Torau
Bukat	Kairiru	Manihiki	Ririo	Tsou
Buli	Kalagan	Manobo, Ata (down river)	Roma	Tugun
Bunun	Kalinga, Limos	Manobo, Dibabawon	Roro	Tunjung
Buol	Kallahan, Keleyqiq	Manobo, Ilianen (Kibudtungan Dialect)	Rotuman	Ubir
Butuanon	Kambera	Manobo, Sarangani, Kayaponga Dialect	Roviana	Ughele
Carolinian	Kanakanabu	Mansaka	Rukai	Ujir (N.Aru)
Cebuano	Kandas	Maori	Rurutuan	Ura
Centra Amis	Kapampangan	Mapun	Sa (south easterndialect)	Uruava
Chamorro	Kapingamarangi	Maranao	Sa'a	Vaeakau-Taumako
Chru	Katingan	Marau	Saaroa	Vaghua
Chuukese (AKA Trukese)	Kaulong (Au Village)	Marovo	Sambal, Botolan	Varisi
Dai	Kavalan	Marshallese	Samoan	Vitu
Dawera-Daweloor	Kayupulau[Kajupulau]	Masiwang	Sangir	Wampar
Dehu	Kazukuru	Matukar	Sasak	Wanukaka
Diodio	Kemak	Maututu	Savu	Waray-Waray
Dorio	Kerinci	Mbaelelea	Seimat	Waropen
Doura	Kilivila	Mbaengguu	Sekar	Watubela
Emae	Kiribati	Mbirao	Selaru	Wedau
Emplawas	Kis	Mekeo	Sengseng	Windesi Wandamen
Ende	Kisar	Mengen	Serili	Wogeo
Erai	Kokota	Minangkabau	Serua	Woleaian
Fagani	Komering	Modang	Siar	Wolio
Fataleka	Kove	Moken	Sika	Wuvulu
Favorlang	Kuni	Molima	Simbo	Yabem
Fijian (Bau)	Kusaghe	Mono	Singhi	Yakan
Futuna-Aniwa	Kusaie	Mota	Siraya	Yamdena
Futuna, East	Kwai	Motu	Soa	Yapese
Gabadi	Kwaio	Nakanai (Bileki Dialect)	Sobei	Zabana (Kia)

Table S8. List of the 305 Austronesian languages considered in both ABVD and ASJP databases. Here we report the complete list of the 305 languages taken in account in our studies of ABVD and ASJP list of words for the Austronesian family. Names of languages are reported as presented in **http://language.psy.auckland.ac.nz/austronesian/.**

—	ABVD			ASJP		
	**Neighbour-Joining**	**FastME**	**FastSBiX**	**Neighbour-Joining**	**FastME**	**FastSBiX**
RF	0.5762	**0.5573**	0.6240	0.6026	0.5993	0.5993
QD	0.3508	0.3657	**0.2371**	0.3605	0.4594	0.3163
GRF	0.5464	**0.5331**	0.5430	0.6192	0.6325	0.5927
GQD	0.2860	0.3024	**0.1609**	0.2969	0.4055	0.2815

Table S9. Accuracy in reconstructing the Austronesian family tree with the ABVD and ASJP database. This table shows the Robinson-Foulds distance, the Quartet Distance and their generalizations, between the Austronesian language tree (inferred with both the ASJP and the ABVD database) and its relative Ethnologue-Classification for the languages shown in Table S8. In this case we only used the LDN definition of distance between lists of words and FastSBiX as distance-based algorithm to infer the trees. All the measures indicate that the ABVD database allows for a more accurate reconstruction of this language tree.

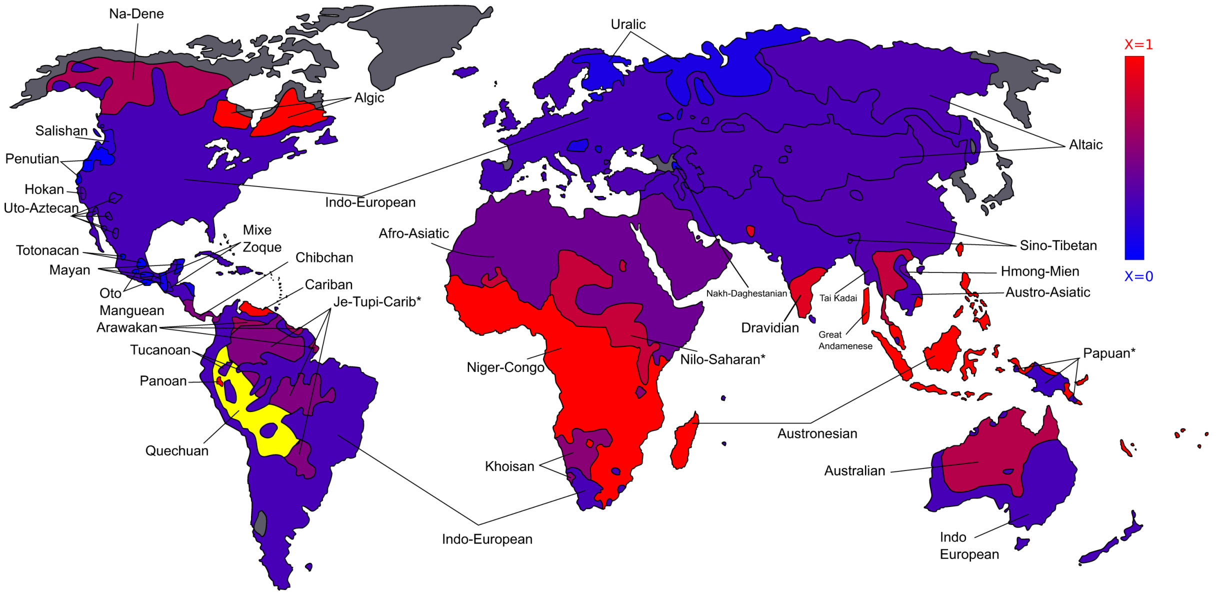

Figure S3. Accuracy of the reconstruction across the planet. FastME, LDN matrix. This map represents the level of accuracy of the FastME algorithm on several language families throughout the world. Trees Inferred with LDN matrices. The legend is the same of Fig. S2.

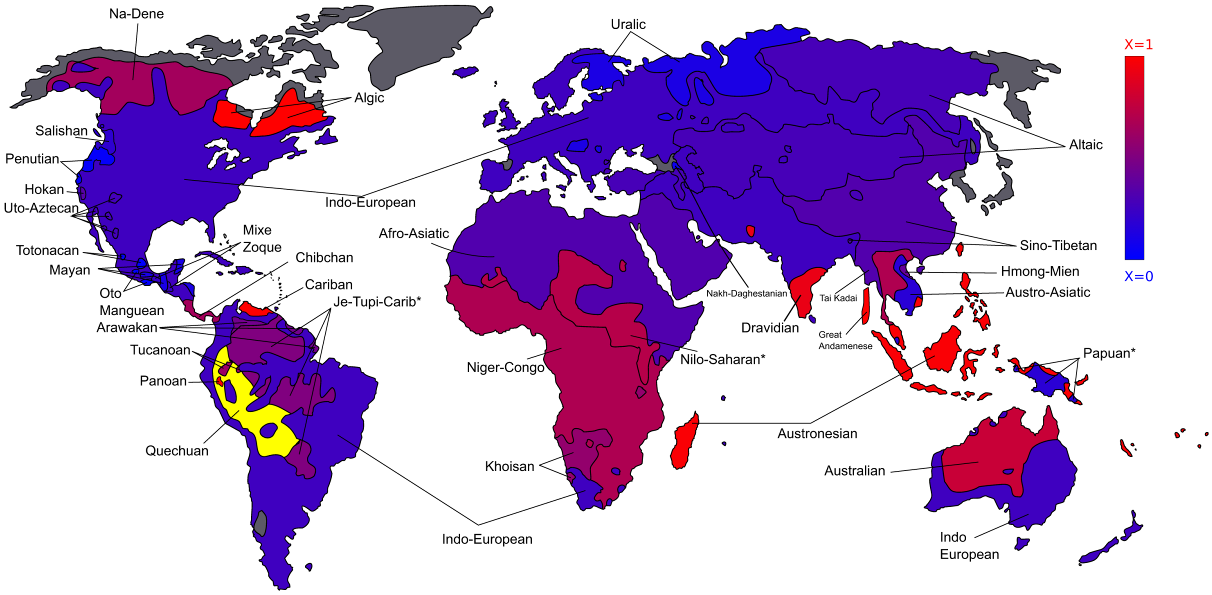

Figure S4. Accuracy of the reconstruction across the planet. FastSBiX, LDN matrix. This map represents the level of accuracy of the Fast-SBiX algorithm on several language families throughout the world. Trees Inferred with LDN matrices. The legend is the same of Fig. S2.

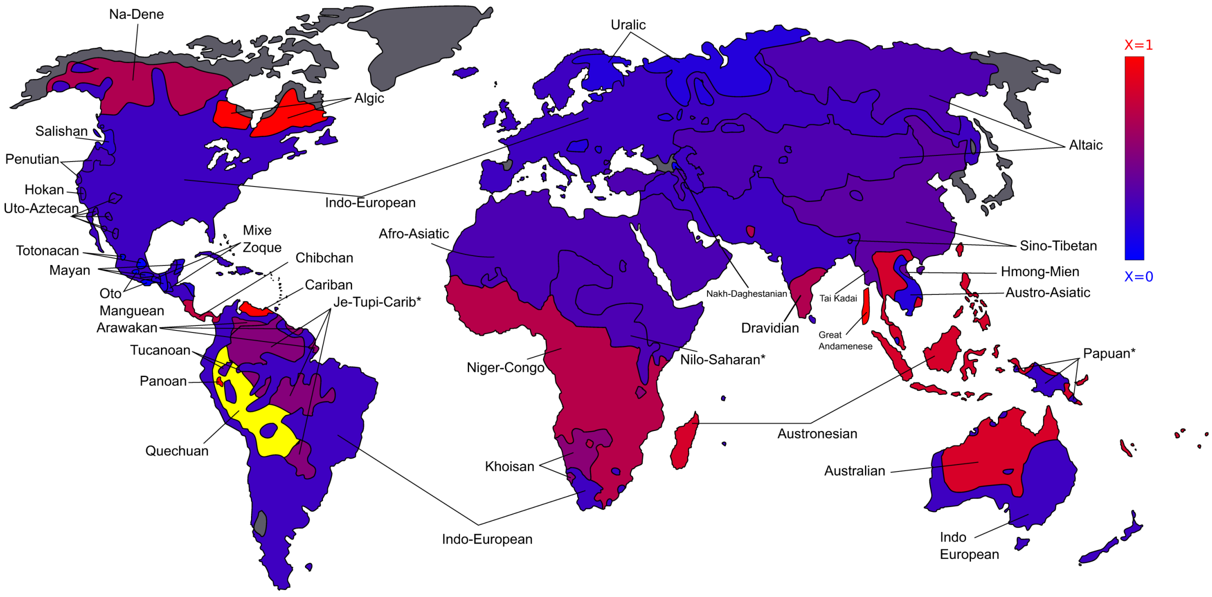

Figure S5. Accuracy of the reconstruction across the planet. NJ, LDND matrix. This map represents the level of accuracy of the Neighbour-Joining algorithm on several language families throughout the world. Trees Inferred with LDND matrices. The legend is the same of Fig. S2.

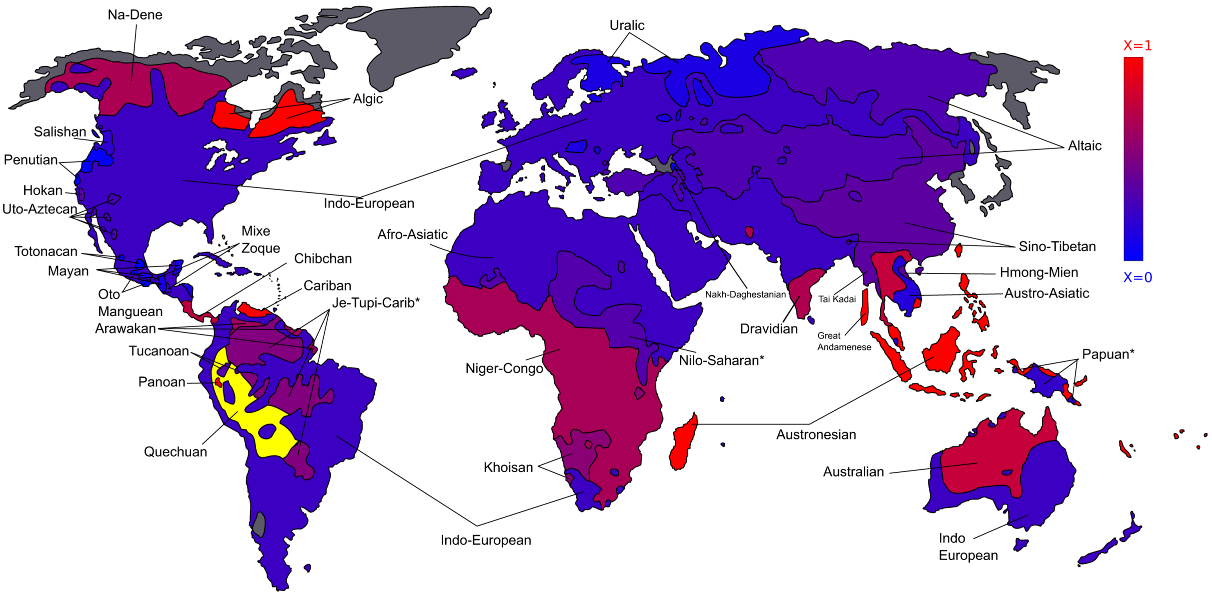

Figure S6. Accuracy of the reconstruction across the planet. FastME, LDND matrix. This map represents the level of accuracy of the FastME algorithm on several language families throughout the world. Trees Inferred with LDND matrices. The legend is the same of Fig. S2.
